# Reassessment of the *Columnea latent viroid* (CLVd) Taxonomic Classification

**DOI:** 10.3390/microorganisms9061117

**Published:** 2021-05-21

**Authors:** Parichate Tangkanchanapas, Annelies Haegeman, Monica Höfte, Kris De Jonghe

**Affiliations:** 1 Plant Sciences Unit, Flanders Research Institute for Agriculture, Fisheries and Food (ILVO), Burgemeester Van Gansberghelaan 96, 9820 Merelbeke, Belgium; parichate_tk@yahoo.com (P.T.); annelies.haegeman@ilvo.vlaanderen.be (A.H.); 2 Department of Plants and Crops, Faculty of Bioscience Engineering, Ghent University, Coupure Links 653, 9000 Ghent, Belgium; monica.hofte@ugent.be; 3 Plant Protection Research and Development Office, Department of Agriculture, Bangkok 10900, Thailand

**Keywords:** pospiviroid, taxonomy, symptom expression, biological characterization, *Solanumstramoniifolium*, *Solanaceae*, multiple sequence analysis, phylogeny

## Abstract

*Columnea latent viroid* (CLVd) is a member of the Pospiviroid family and its naked circular RNA genome typically forms native “rod-like” secondary structures. In this work, the CLVd taxonomy was reevaluated based on sequence similarity and phylogenetic analysis, as well as the evaluation of the symptom development and disease severity of four selected CLVd isolates in a range of host species. The phylogenetic analysis showed that all CLVd isolates were clustered into five distinct clades: (I) severe isolates originally found in tomato crops in Thailand, (II) ornamental isolates, (III) mild isolates originally found in tomato crops in Thailand, and two clades (IV and V) containing mild isolates originating mainly from tomato crops in European countries, with different virulence levels on several hosts. Our analysis demonstrated that some CLVd isolates have a sequence similarity of less than 90% within the species taxon, as well as distinct biological characteristics (symptom development and virulence), both of which are important ICTV criteria for viroid classification. For these reasons, we propose that CLVd should be re-classified into at least three main taxonomic lineages: a “CLVd-tomato Asian lineage” (I), a “CLVd-tomato European lineage” (IV) and a “CLVd-ornamental European lineage” (II), plus two minor lineages (III and V), fitting the ICTV criteria.

## 1. Introduction

Viroids are the smallest plant pathogens and are composed of closed circular single-stranded RNA [1]. Unlike viruses, viroids have no protein capsid and do not possess any protein coding sequences [2]. *Columnea latent viroid* (CLVd) is one of the Pospiviroid family members that forms native “rod-like” secondary structures. It was first isolated from an asymptomatic lipstick vine (*Columnea erythrophae*) in the state of Maryland, USA [3]. The host range of CLVd is mainly limited to members of the Solanaceae family, such as tomato (*Solanum lycopersicum*), potato (*Solanum tuberosum*), eggplant (*Solanum melongena*), chili pepper (*Capsicum annuum*), petunia (*Petunia×hybrida*), bolo maka (*Solanum stramoniifolium*), gynura (*Gynura aurantica*), *Brunfelsia undulata*, and in addition, edible chrysanthemum (*Glebionis coronaria*), ornamentals (*Nematanthus wettsteinii*, *Gloxinia gymnostoma*, *G. nematanthodes*, *G. purpurascens*) and cucumber (*Cucumis sativus*) [3,4,5,6,7,8,9,10]. CLVd is mainly transmitted in a mechanical manner and through seeds [4,6,11,12,13]. Symptoms caused by CLVd are very similar to other pospiviroids, and include stunting, mostly observed on several *Solanum* spp. In susceptible hosts, distortion and vein necrosis symptoms can be found on several plant parts, such as the stem, branch and mid leaf. This viroid can cause a reduction in fruit size and immature seed production in tomatoes [5,13,14,15]. CLVd does not cause symptoms in most ornamental host plants [8,9,10].

Viroid classification and nomenclature is managed by the International Committee on Virus Taxonomy (ICTV) and is based on molecular features (e.g., rod-like conformations, the type of central conserved region (CCR), the presence or absence of a terminal conserved region (TCR), etc.), as well as two additional mandatory criteria—an arbitrary level of less than 90% sequence similarity to other related viroid species, and distinct biological properties, particularly host range and symptoms [16,17,18]. The current taxonomy of CLVd is not completely consistent with these main taxonomic criteria. Firstly, CLVd most likely arose from an RNA recombination (Hammond et al. 1989) and even if it is classified within the genus *Pospiviroid*, it shares its CCR and TCR with the members of the *Hostuviroid* genus. In addition, within this taxonomic species, some CLVd isolates have a sequence similarity of less than 90% within the species taxon, especially when Asian and European isolates are compared, as we show in this study. Moreover, some biological properties within this species, such as symptom severity, are quite different between isolates. Ever since CLVd was found in South East Asia in tomato production crops, very severe symptoms and high crop losses have been observed and reported, particularly in Thailand. The Thai CLVd-infected tomatoes showed very severe stunting, strong leaf rugosity and strong vein necrosis [5,13,14,19,20], combined with very high yield losses (around 50% reduction in fruit size) in several commercial tomato cultivars [13]. On the other hand, mild symptoms and much less crop losses have been reported from most of the European CLVd isolates [12,21,22]. Since the ninth report, only one CLVd strain, designated as CLVd-col (*Columnea latent viroid*-*Columnea* isolate) has been recognized by ICTV [16,21], whereas previously three separate strains, namely, CLVd-bru (*Brunfelsia undulata*), CLVd-nem (*Nematanthus wettsteinii*) and CLVd-col, were assigned to the CLVd species [8,9,21,23].

In contrast, several other viroids have been recognized as distinct isolates by ICTV in both the Pospiviroidae and Avsunviroidae families due to differences in biological characteristics such as host range and symptoms [16]. However, they still have over 90% genome sequence similarity, and hence can still be considered the same species. For instance, *Citrus exocortis viroid* (CEVd) is assigned into two host-types; CEVd—citrus and CEVd—tomato (Indian tomato bunchy top viroid). Two host-types have been assigned for *Potato spindle tuber viroid* (PSTVd), namely, PSTVd—intermediate and PSTVd—tomato. Interestingly, *Hop stunt viroid* (HpSVd, currently HSVd) was split into five “species”, linked to the host-types in the previous (ninth) ICTV report; HpSVd—hop, HpSVd—citrus HpSVdcucumber (*Cucumber pale fruit viroid*), HpSVd—peach (*Peach dapple viroid*) (HpSVd-pch) and HpSVd—plum (*Plum dapple viroid*) [16].

In this study, the taxonomy of the currently known CLVd isolates is re-evaluated based on sequence similarity and phylogenic tree analysis, as well as taking into account the symptom development and disease severity across several Solanaceae hosts, such as tomatoes, bolo maka, Thai round eggplant, chili pepper, bell pepper (*Capsicum annuum*) and cucumber. Based on our findings, we propose re-designating the CLVd taxonomic classification into at least three distinct lineages (or strains), with most Asia-related CLVd isolates belonging to a “CLVd-tomato Asian lineage”, the European tomato isolates as a “CLVd-tomato European lineage” and the European ornamental isolates as a “CLVd-ornamental European lineage”.

## 2. Materials and Methods

### 2.1. Characterization of CLVd’s Biological Properties

#### 2.1.1. Plant and Viroid Maintenance

Two-week-old tomato (cvs. Rutgers, Seeda 50 and Insaf) and cucumber plants, and three-week-old bolo maka, Thai round eggplant, chili pepper and bell pepper plants (3–5 plants per host) were mechanically inoculated (slashing) with four CLVd isolates—14A, Chaipayon-1, LP1-15c4 and Solanum-1 (Table 1). In addition, mock inoculations were performed as a negative control. Plants were kept in a greenhouse at 27 °C with a 17 h light/7 h dark light regime. In all inoculated plants, viroid infection was confirmed by means of RT-PCR four to six weeks after inoculation, depending on the plant species. Symptoms were observed and recorded and disease severity for the infected plants was assessed as explained below.

#### 2.1.2. RT-PCR

CLVd infection was confirmed using CLVd-specific (c-CLVd-infect: TGCAGGGTCAGGTGTGAACCAC and h-CLVd-infect: GCCATGCAAAGRAAAAAGAAYGGG) [24] and two generic pospiviroid RT-PCR detection tests [14,25]. Total RNA was extracted from 100 mg fresh leaves from viroid-infected plants using the Spectrum™ Plant Total RNA Kit (Sigma-Aldrich, Saint Louis, MI, USA) following the manufacturer’s instructions.

cDNA was synthesized by means of the iScript™ cDNA Synthesis kit (Bio-Rad, Hercules, CA, USA). PCR reactions were performed using the FastStart™ Taq DNA Polymerase (Sigma-Aldrich, Saint Louis, MI, USA) under conditions described in the respective publications. All steps were performed according to the respective manufacturer’s protocols in a T100™ Thermal Cycler (Bio-Rad, Hercules, CA, USA). The RT-PCR amplification products were analyzed by means of gel electrophoresis using a 2% agarose gel, and Midori Green Direct as the staining dye (Nippon Gene Co., Ltd., Düren, Germany). The results were visualized under blue light using an Azure c150 Gel Imaging Workstation (Azure Biosystems, Inc., Dublin, CA, USA). Again, all steps were performed according to the manufacturer’s protocols. Subsequent Sanger sequencing of the amplicon was performed, outsourced at Macrogen (Amsterdam, The Netherlands) and after contig assembly in Bionumerics (Bionumerics, version 7.6.3; Bioinformatics software; Applied Maths: Sint-Martens-Latem, Belgium, 2020), a BLASTn search against the non-redundant Nucleotide database (https://blast.ncbi.nlm.nih.gov/Blast.cgi, accessed on 29 January 2018) was used to confirm the viroid identity.

#### 2.1.3. Symptoms and Severity Rating

CLVd-infected plants were photographed, disease severity was assessed and symptoms were observed and recorded using symptom rating scale tables (Table 2 and Table 3). The symptom rating scale was calculated by scoring the main typical symptoms, multiplied by a weight score (based on damage and the severity of the symptoms). Five dominant symptoms—yellowing, necrosis, distortion and stunting of apical shoot and stem—were used for symptom rating on tomato plants (Table 2). For bolo maka, Thai round eggplant, chili/bell pepper and cucumber, only three main symptoms, namely, leaf distortion, necrotic lesion and stunting, were scored and assessed (Table 3). Each of the selected and rated symptoms were ranked into a scale containing six levels (from 0 (no symptoms) to 5 (most severe)). The symptom rating scores from each selected host were averaged for all plants (3 to 5 plants per host). The score x weight was calculated based on “the score” multiplied by the “weight of score” (of each symptom). The overall symptom rating scale was calculated using the equation presented below. The overall symptoms ranged from 0 to 5 and an average score of 0–1 was indicated as “mild”, a score 2–3 was “intermediate”, and a score of 4–5 was “severe”.

(1) $$\mathrm{Average}\;\mathrm{overall}\;\mathrm{symptom}\;\mathrm{rating}\;=\;\frac{\sum (\mathrm{average}\;\mathrm{score}\;\mathrm{for}\;a\;\mathrm{specific}\;\mathrm{symptom}\;\times \;\mathrm{average}\;\mathrm{score}\;\mathrm{weight}\;\mathrm{for}\;\mathrm{that}\;\mathrm{symptom})}{100}$$

### 2.2. CLVd Genome Sequence Similarity Analysis

#### 2.2.1. CLVd Sequence Analysis

In total, 83 sequences of the *Columnea latent viroid* (CLVd) from the NCBI GenBank database (https://www.ncbi.nlm.nih.gov/, accessed on 30 January 2020) (Table A1) were selected for multiple-sequence alignment and phylogenic tree analysis. For all these CLVd sequences, a multiple-sequence alignment was created with MUSCLE (MEGA7, version 7.0.21, Molecular Evolutionary Genetics Analysis software, Kumar, Stecher, Li, Knyaz, and Tamura: 2018) using default parameters [26]. The phylogenetic tree was created in MEGA7 using neighbor-joining (bootstrap 1000 replicates). The alignment was read into R v4.0.3 (R, version 4.0.3, A language and environment for statistical computing, R Core Team: Vienna, Austria, 2021) using the Biostrings package, and pairwise similarities between the sequences were calculated using the Jukes-Cantor 69 model (assuming equal base frequencies) in the phangorn package. Finally, the sequences were clustered based on their pairwise similarity using hierarchical clustering, and the similarities were visualized in a heatmap using the gplots package.

#### 2.2.2. CLVd Symptom Severity and Sequence Similarity Analysis

The results of the symptom severity assessment for the four selected CLVd isolates (2.1) were evaluated with respect to the CLVd genome sequence similarity analysis from 83 CLVd isolates. A pairwise identity matrix (%) of four selected CLVd isolates was created using Clustal2.1 (https://www.ebi.ac.uk/Tools/msa/clustalo/, accessed 30 January 2020).

## 3. Results

### 3.1. Characterization of CLVd’s Biological Properties

#### 3.1.1. Phylogenetic Analysis of Four Selected CLVd Isolates

**Figure 1:**
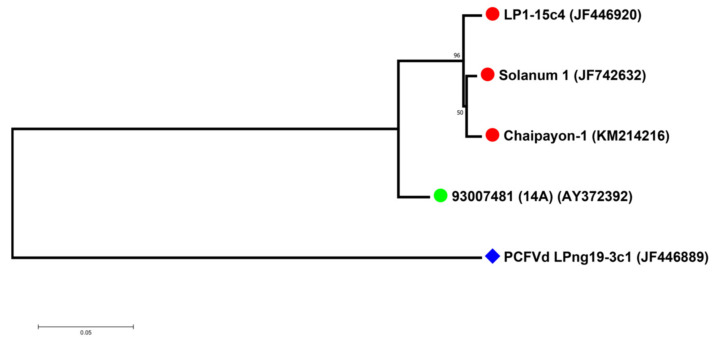
Phylogenetic relation between four CLVd isolates. The European CLVd isolate is indicated with a green dot, whereas the Thai CLVd isolates are indicated with a red dot. PCFVd LPng19-3c1 (GenBank Accession No. JF446920) was used as an outgroup and is indicated with a blue diamond shape. The tree was constructed using MEGA version 7.0.21 with 1000 bootstrap replicates and a 50% cut-off value.

The phylogenetic analysis of the sequences of the four selected CLVd isolates (14A, Chaipayon-1, LP1–15c4 and Solanum-1) showed that the three Thai CLVd isolates clustered into the same clade, separately from the European isolate (Figure 1). The percentage identity matrix showed the distinction between European and Thai isolates, close to the taxonomic species-threshold of 90% (Table 4).

#### 3.1.2. Symptoms and Severity Rating

##### Tomato cv. Rutgers

**Figure 2:**
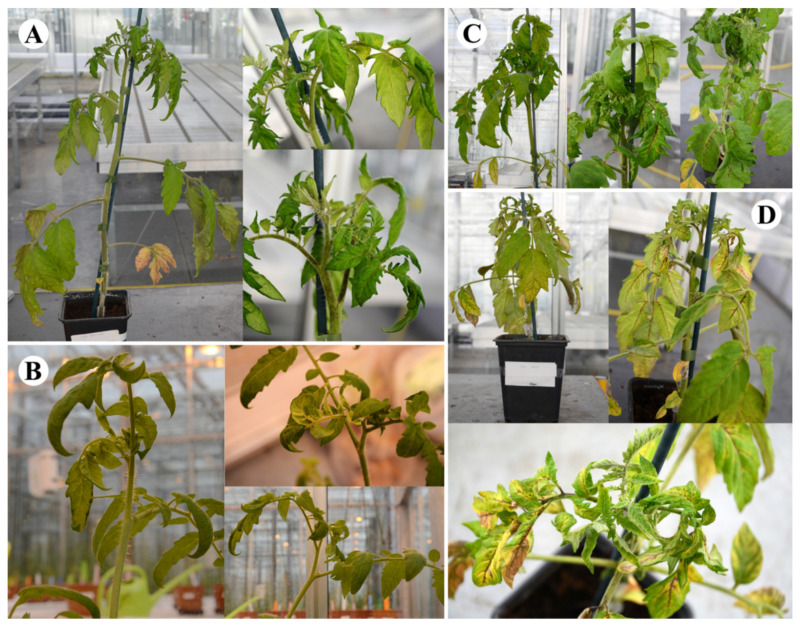
Symptoms on tomato cv. Rutgers of four selected CLVd isolates; 14A, Chaipayon-1, Solanum-1 and LP1-15c4 (**A**, **B**, **C** and **D**, respectively).

All four CLVd isolates induced typical viroid symptoms on tomato cv. Rutgers, such as strong vein necrosis, leaf rugosity and epinasty and severe stunting. CLVd 14A showed the least symptoms compared to the three Thai isolates (Figure 2). A score of 3.2 on the symptom rating scale was recorded for CLVd 14A, inducing intermediate symptoms in tomato cv. Rutgers (Table 5). CLVd Chaipayon-1 showed more severe symptoms (stunting, epinasty, rugosity and necrosis) than CLVd 14A, and scored an average of 4.8 on the symptom rating scale (Figure 2, Table 5 and Table 6), whereas both CLVd LP1-15c4 and Solanum-1 resulted in the most severe symptoms in tomato cv. Rutgers. These two CLVd isolates induced extreme stunting of the whole plant, whereas leaves showed strong yellowing, epinasty and rugosity. Strong obvious necrotic lesions were found in several plant parts, such as the midrib, leaf vein, petiole and stem (Figure 2). Both isolates scored an average of 5.0 on the symptom rating scale (Table 5).

##### Tomato cv. Seeda 50

**Figure 3:**
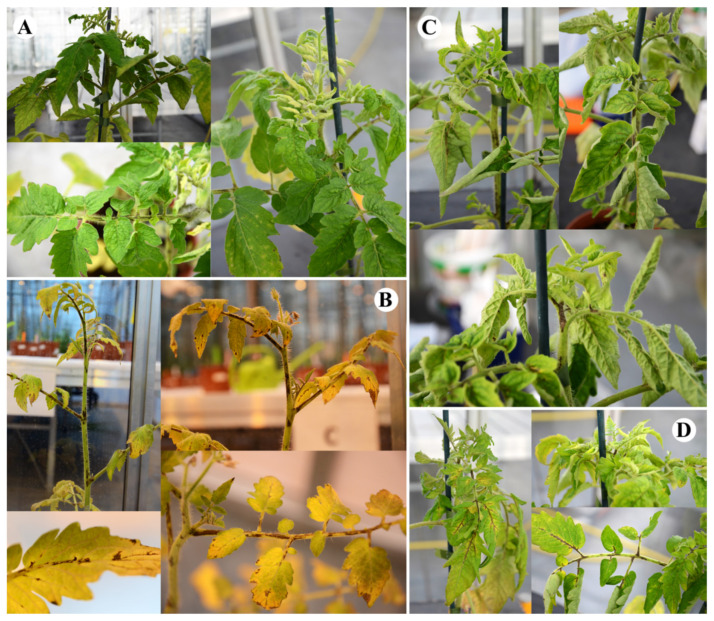
Symptoms on tomato cv. Seeda 50 of four selected CLVd isolates; 14A, Chaipayon-1, Solanum-1 and LP1-15c4 (**A**, **B**, **C** and **D**, respectively).

For tomato cv. Seeda 50, the overall symptoms were quite similar, although generally less severe than in the cv. Rutgers. Notably, bunchy top symptoms were less pronounced in this tomato cultivar. Again, CLVd 14A (score 2.6) showed the mildest symptoms compared to all Thai isolates, mostly inducing moderate stunting and necrotic lesions, although it showed less yellowing, epinasty and rugosity (Figure 3, Table 5 and Table 6).

CLVd Chaipayon-1 (score 3.8) caused extensive yellowing and necrosis in the whole plant; however, less epinasty and rugosity was observed. (Figure 3, Table 5 and Table 6). Furthermore, on cvs. Seeda 50, CLVd LP1-15c4 and Solanum-1 induced the strongest symptoms, with again yellowing of leaves, very severe necrosis and stunting, as well as slightly less epinasty and rugosity compared to Rutgers (Figure 3 and Table 6), which had a score of 4.5 (severe) for both CLVd isolates (Table 5).

##### Tomato cv. Insaf

**Figure 4:**
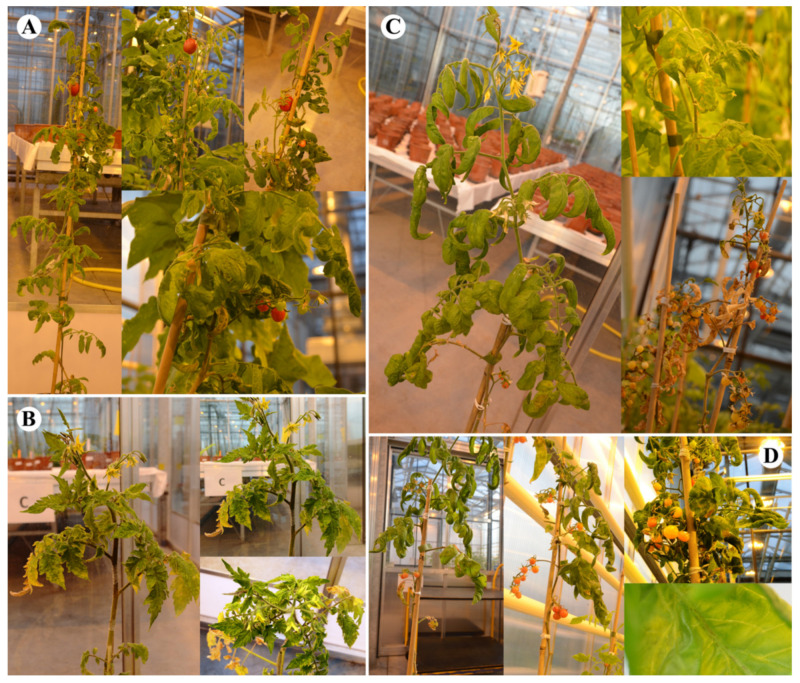
Symptoms on tomato cv. Insaf of four selected CLVd isolates; 14A, Chaipayon-1, Solanum-1 and LP1-15c4 (**A**, **B**, **C** and **D**, respectively).

For tomato cv. Insaf, the overall symptoms were less severe than for cv. Seeda 50 and tomato cv. Rutgers, and again, CLVd 14A (score 1.1) showed the mildest symptoms. On cv. Insaf, only very mild stunting, yellowing, epinasty, rugosity and necrosis symptoms were observed (Figure 4, Table 5 and Table 6).

CLVd Chaipayon-1 (score 2.8) caused stronger typical symptoms compared to CLVd 14A, with obvious stunting, yellowing, leaf epinasty and rugosity, as well as necrosis on the whole plant (Figure 4, Table 5 and Table 6). CLVd LP1-15c4 and Solanum-1 induced similar symptoms, with apical stunting and leaf yellowing, epinasty and rugosity, yet only limited stem stunting and necrosis, resulting in a similar score for both isolates (Figure 4, Table 5 and Table 6).

##### Bolo Maka

**Figure 5:**
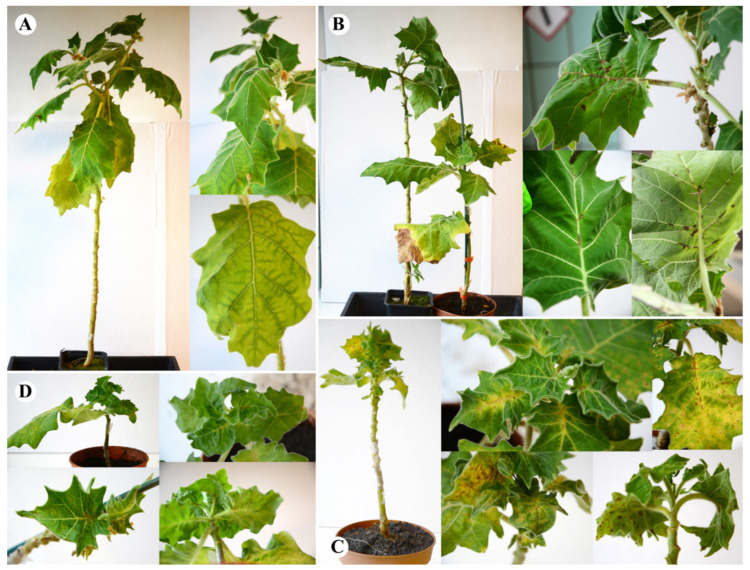
Symptoms on bolo maka of four selected CLVd isolates; 14A, Chaipayon-1, Solanum-1 and LP1-15c4 (**A**, **B**, **C** and **D**, respectively).

In bolo maka, a high variation in symptom development and severity was observed. Generally, symptoms on bolo maka were quite similar to those observed on the tomato cultivars. The plants showed stunting of the whole plant, leaf epinasty, rugosity, yellowing and apical stunting. Similarly as observed in tomatoes, CLVd 14A induced the least symptoms, compared to the three Thai isolates. The isolate only induced slight growth reduction and very mild leaf distortion (epinasty and rugosity). No necrosis was observed in any of the infected bolo maka plants (Figure 5 and Table 6). CLVd Chaipayon-1 caused stronger symptoms, especially leaf epinasty and rugosity with strong vein necrosis on the leaf vein, midrib, petiole and stem. CLVd Chaipayon-1 caused more severe stunting than CLVd 14A (around a 30% reduction in plant height) (Figure 5 and Table 6). CLVd LP1-15c4 and Solanum-1 induced the most severe symptoms; including very strong stunting (more than a 70% reduction in plant height), severe leaf distortion showing epinasty, rugosity and size reduction. Vein necrosis was more distinct than for CLVd Chaipayon-1 (Figure 5 and Table 6). The obtained disease severity scores for these four CLVd isolates are presented in Table 5.

##### Thai Round Eggplant

In Thai round eggplant, generally the typical symptoms were much less severe than in tomatoes and bolo maka. The most typical symptoms that were observed were overall mild stunting and yellowing, with only slight leaf epinasty and rugosity. In addition, necrotic lesions were rarely found, and if so, then it was particularly for the more severe isolates CLVd LP1-15c4 and Solanum-1, and this occurred late in the infection period. CLVd 14A caused almost no symptoms on Thai round eggplant. Only a minor leaf distortion was observed. CLVd Chaipayon-1 also caused fewer symptoms than CLVd 14A. The symptoms largely remained limited to a minor reduction in plant height. CLVd LP1-15c4 and Solanum-1 showed slightly stronger symptoms than CLVd 14A and Chaipayon-1 and caused leaf distortion and slight vein necrosis on the midrib, petiole and stem (Table 6). The obtained disease severity scores for these four CLVd isolates are presented in Table 5.

##### Chili Pepper, Bell Pepper and Cucumber

There was no apparent symptom development on chili or bell pepper for any of the CLVd isolates (Table 6). On the other hand, all of the infected (chili and bell) pepper plants resulted in a very strong positive band in the RT-PCR. In addition, the CLVd infection remained latent for the whole life span of the plants, also not inducing symptoms on flowers and fruits. No effect on seed development was observed. For cucumber, only CLVd Solanum-1 succeeded in mechanically infecting the cucumber plants and RT-PCR results confirmed an infection rate of 50% (3/6 plants). None of the infected cucumber plants developed symptoms (Table 6).

##### Severity Evaluation of Four CLVd Isolates

**Figure 6:**
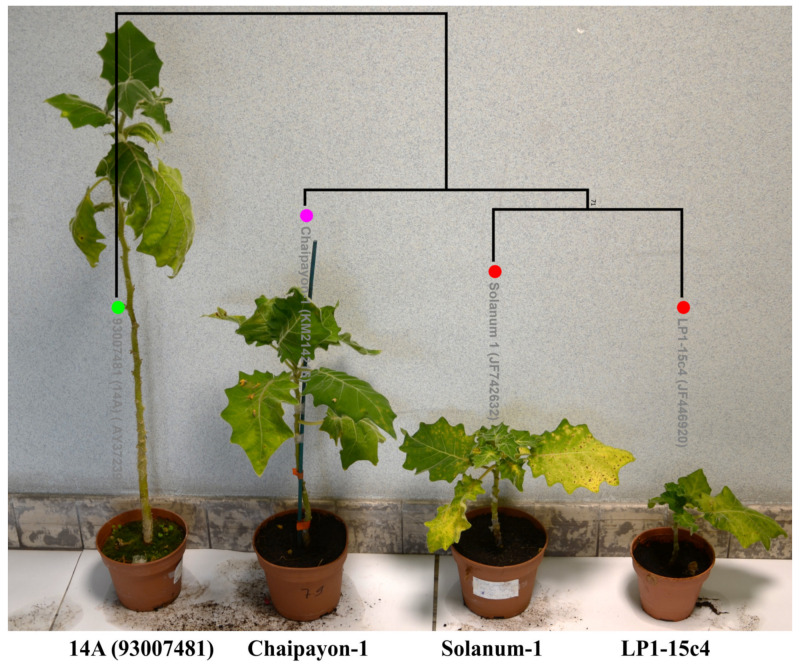
Severity and symptoms comparison of four CLVd isolates on bolo maka (7 months after inoculation). The symptom severity with respect to stunting was compared and evaluated as mild (14A), intermediate (Chaipayon-1) or severe (LP1-15c4 and Solanum-1) for the four included isolates in the study. The phylogenetic tree analysis is shown on top (the tree was constructed using MEGA version 7.0.21 with 1000 bootstrap replicates and 50% cut-off value).

Overall, CLVd 14A was the mildest of all four selected isolates in this study. It showed intermediate symptoms on tomato cvs. Rutgers and Seeda 50, whereas it caused mild symptoms on tomato cv. Insaf, bolo maka and Thai round eggplant (Table 5 and Table 6 and Figure 6). CLVd Chaipayon-1 can be considered as an isolate inducing intermediate symptoms. Although it did result in quite severe symptoms on tomato plants, more intermediate symptoms were observed on bolo maka, and its infection remained rather mild on Thai round eggplant (Table 6). Because the symptoms of CLVd Chaipayon-1 were generally less severe than those of the isolates CLVd LP1-15c4 and Solanum-1, this CLVd isolate was considered to be intermediately virulent (Figure 6 and Table 6). Finally, the highly virulent CLVd LP1-15c4 and Solanum-1 isolates resulted in an almost identical severity. These two isolates caused extreme symptoms on tomato cvs. Rutgers and Seeda 50 as well as on bolo maka, whereas they induced intermediate symptoms in tomato cv. Insaf and remained rather mild in Thai round eggplant (Table 5 and Table 6 and Figure 6).

Phylogenetic analysis showed that the sequence similarity of the four selected CLVd isolates is correlated with the symptom severity that they induce. Of the three Thai CLVd isolates, the two severe isolates (LP1-15c4 and Solanum-1) were the most closely related, yet they also clustered with the intermediate isolate (Chaipayon-1), whereas the CLVd 14A mild isolate was completely segregated from the others (Figure 6).

### 3.2. CLVd Genome Sequence Similarity Analysis

#### 3.2.1. CLVd Sequence Analysis

**Figure 7:**
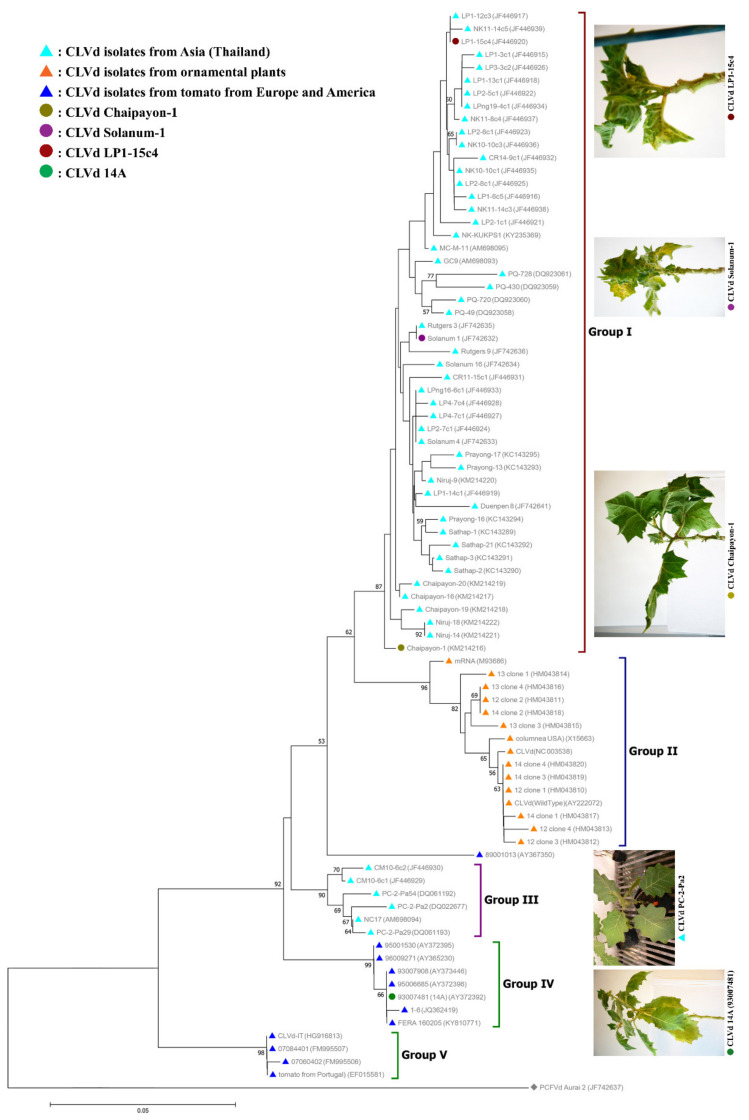
Neighbor-joining phylogenetic tree analysis of all CLVd genome sequences from GenBank. The tree was constructed using MEGA version 7.0.21 with 1000 bootstrap replicates and a 50% cut-off value. *Pepper chat fruit viroid* (PCFVd) isolate Aurai 2 (accession no. JF742637) was used as an outgroup. Symptom severity of four selected CLVd isolates and CLVd PC-2-Pa29 [14,19], causing mild symptoms, were compared.

The phylogenetic tree (Figure 7), created based on the full genome sequences of all CLVd isolates from GenBank, shows that five distinct clades, or groups, can be differentiated—group I mainly contains tomato isolates from Asia (represented with a light blue triangle), except isolates Solanum-1, Solanum-4 and Solanum-16, which were obtained from bolo maka. Most isolates from this clade induce severe symptoms on tomatoes. All three selected CLVd isolates (Chaipayon-1, Solanum-1 and LP1-15c4) also group into this clade.

Group II includes all ornamental isolates (represented with an orange triangle). This clade consists of isolates from European countries, plus three isolates from the United States and a Canadian isolate.

Group III contains a minor group of tomato isolates from Asia (Thailand) that cluster distantly from the major Asian tomato isolate group (I). The isolates in this group were all obtained during the very first CLVd outbreak in Thailand, which was eradicated, since related variants have never been found again in the country.

Group IV consists of tomato isolates from Europe (represented with a dark blue triangle), mainly originating from the Netherlands and the United Kingdom (GenBank Accession No. KY810771), plus one isolate from Mali. One of the selected isolates in this study, CLVd-14A, originally found in tomato crops in the Netherlands, clusters in this group.

Finally, group V contains some remaining deviating minor tomato isolates from Europe that were collected in France, Portugal and Italy.

**Figure 8:**
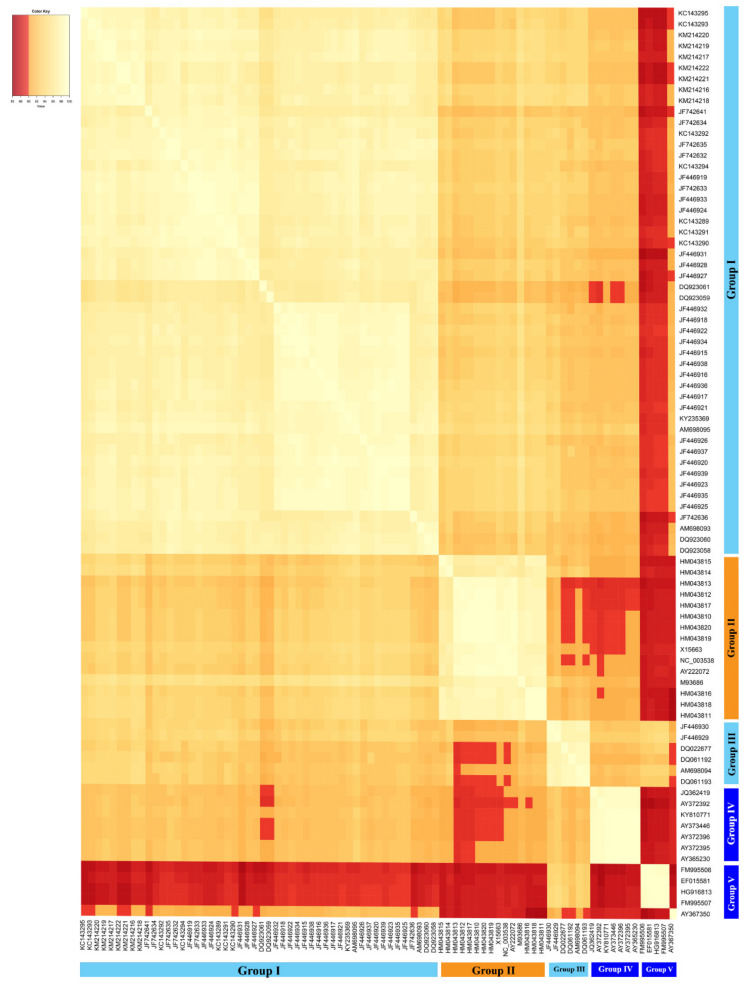
Multiple-sequence alignment matrix of 83 CLVd isolates. The CLVd genome sequences clustered into two main groups. Group V isolates show a sequence similarity of less than 90% with the other isolates. The color scale represents a level of sequence similarity ranging from yellow (90%) to white (100%). The red color blocks indicate a level of sequence similarity less than 90%.

A multiple-sequence similarity matrix was created based on the 83 publicly available complete sequences of CLVd isolates and visually presents the pairwise genome sequence similarity within the viroid species “*Columnea latent viroid*”. The genome sequences of CLVd showed the variation in sequence similarity across this viroid species and also reflect the five groups that were discussed above (Figure 8). The CLVd genome sequence similarity analysis (one of two mandatory viroid taxonomic criteria) also reveals that most CLVd isolates, particularly those belonging to CLVd group V, show a sequence similarity of less than 90% (indicated with a red color in Figure 8) with isolates of the other four groups (Figure 8). This is quite atypical for virus and viroid taxonomy, since the level of sequence similarity needs to be greater than or equal to 90% in order for isolates to be classified as members of the same species.

#### 3.2.2. CLVd Symptom Severity and Sequence Similarity Analysis

All viroids from group I induced clearly different symptoms and severity, especially in several Solanaceae hosts, compared to, e.g., CLVd 14A, the representative isolate from group IV that was intensively studied. They are highly virulent and cause important yield losses in tomato crops and bolo maka [5,14,15,19]. The symptom severity of three selected CLVd isolates (Chaipayon-1, Solanum-1 and LP1-15c4) of this group was demonstrated and discussed in Section 3.1.2.

Isolates from group II, the ornamental European lineage, were previously reported not to cause any symptoms on their (original) natural ornamental host species; *Columnea erythrophae*, *Nemathanthus wettsteini*, *Brunfelsia undulate*, *Gloxinia gymnostoma*, *G. nematanthodes* and *G. purpurascens* [8,9,10,12]. Isolates from this CLVd group induced mild to intermediate symptoms on tomato plants. They caused symptoms on tomato and potato crops similar to those of other pospiviroids; however, they proved to be less severe than the highly virulent isolates of potato spindle viroid (PSTVd) [12].

Isolates from group III, the minor tomato Asia lineage, induce mild to intermediate symptoms on several Solanaceae hosts (Figure 7). They only induced mild to intermediate symptoms on tomato plants, and almost no symptoms on bolo maka and Thai round eggplant [14,19] This is again in contrast with the isolates from group I, the major tomato lineage from Asia, inducing severe symptoms on all Solanaceae vegetable hosts such as tomato, bolo maka and Thai round eggplant [5,15,19] and intermediate symptoms on Thai round eggplant and hot chili [5,14,19].

CLVd 14A perfectly clusters in CLVd group IV, the major tomato European lineage, and was originally found in a tomato plant in the Netherlands. Group IV isolates generally show mild to intermediate symptoms on several plant species and induce typical pospiviroid symptoms on tomato and potato plants [7,27]. Only one observation of a CLVd outbreak in UK has been recorded, which showed no symptoms on the fruit and no obvious negative effects on quality [28].

The minor tomato European lineage, group V, also induced viroid-like symptoms on tomato crops—stunting, vein necrosis and leaf yellowing and malformation [29,30]. 

The isolates from group I showed a sequence similarity lower than 90% with CLVd group V and some members in group IV. They also share a relatively low sequence similarity with isolates from CLVd group II and III, at 93.2% and 92.8% on average, respectively. In addition, isolates from this group clearly induced distinct symptoms on bolo maka and yield loss in tomatoes, when compared to CLVd group II, III and IV. However, it is impossible to discuss the biological properties of CLVd group V, because no data about symptom expression on bolo maka and yield loss in tomatoes of this group have been reported. Furthermore, the isolates from group II, the ornamental lineage, showed less than 90% sequence similarity to members of the CLVd group V, and some isolates from group III and IV. On average, they shared 93.2% sequence similarity with the isolates in CLVd group I. This CLVd lineage induced mild to intermediate symptom virulence in tomatoes and almost no symptoms on bolo maka, which is similar to CLVd group III and IV. Group III, the minor tomato Asia lineage, showed less than 90% sequence similarity to some members of CLVd group II, and shared low sequence similarity to CLVd groups I, IV and V (92.8%, 91.8% and 92.5% on average, respectively). Group IV, the major tomato European lineage, showed less than 90% sequence similarity to CLVd group V and some members of CLVd groups I and II. It shared on average only 91.8% sequence similarity to CLVd group III. Group V, the minor tomato European lineage, showed less than 90% sequence similarity to CLVd groups I, II and IV, whereas it shared on average 92.5% sequence similarity to CLVd group III (Figure 8).

#### 3.2.3. Conserved Motifs along the CLVd Genome

**Figure 9:**
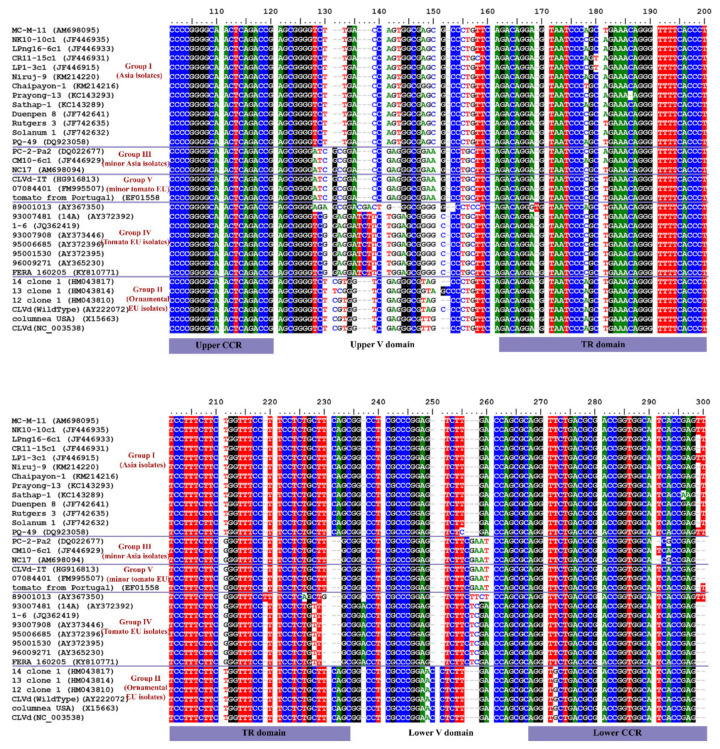
Conserved motifs of CLVd isolates in the upper and lower V domain. CLVd genome sequence multiple alignment analysis of the five selected CLVd groups.

The CLVd genome sequence multiple alignment analysis showed particular variations of conserved motifs in the variable (V) domain between the different CLVd groups. In detail, most CLVd groups showed a distinct sequence conserved motif in the upper V domain, except CLVd groups III and V (minor Asia and minor tomato European groups) which both contain an identical sequence motif (Figure 9). In the lower V domain, most CLVd groups show differences in the conserved motif as well. The main tomato European lineage (group IV) contains a distinct conserved motif, whereas in the main Asian (group I) and the ornamental European lineage (group II), a similar conserved motif can be observed. Furthermore, CLVd groups III and V contain an almost identical sequence in the lower V domain as well (Figure 9). This may suggest that the minor Asia lineage (group III) and minor tomato European lineage (group V) are more related to each other than to the other CLVd groups, and that they share a similar evolutionary route. On the other hand, the major Asian (group I), ornamental European (group II) and major tomato European (group IV) lineages contain a totally different conserved motif at the V domain, which suggests a dissimilar evolutionary route.

## 4. Discussion

In this work, we investigated the virulence of four *Columnea latent viroid* (CLVd) isolates (Chaipayon-1, Solanum-1, LP1-15c4 and 14A) on different host plants, revealing clear variations in symptom severity. The imperfect current taxonomic classification of *Columnea latent viroid* species was studied using genome sequence similarity analysis of publicly available CLVd sequences and reevaluated in light of some biological properties, such as symptom expression and virulence.

CLVd is a poorly studied member of the pospiviroids, and information about its taxonomy, host range, transmission methods and impact are limited [21]. To date, only one research article has proposed re-classifying CLVd’s taxonomy (from *Pospiviroid* into the *Hostuviroid* genus) based on high-throughput selective 29-hydroxyl acylation analyzed by primer extension (SHAPE) and RNA structure prediction software. However, in this study, only one CLVd reference isolate was used (GenBank Accession No. AY367350, including in CLVd group V) [31].

Viroid pathogenicity and disease severity are directly related to the viroid genome sequence, in combination with the host plant species and age, as well as environmental factors (e.g., light intensity and ambient temperature) [32,33]. Four different CLVd isolates were selected to evaluate symptom development and disease severity across a range of plant species—tomato cvs. Rutgers, Seeda 50 and Insaf; bolo maka; Thai round eggplant; chili pepper; bell pepper and cucumber. CLVd 14A, isolated from tomatoes in Europe, proved to be a very mild isolate, overall causing much milder symptoms in all selected plant species than the three Thai isolates [7,28]. The intermediate isolate CLVd Chaipayon-1, found on tomato plants in the northeastern region of Thailand [5,19], induced strong symptoms only on tomato cvs. Rutgers and Seeda 50, while causing moderate symptoms on tomato cv. Insaf and bolo maka. Lastly, the severe isolates CLVd LP1-15c4 and Solanum-1 showed very strong symptoms in most of the plant species; tomato cvs. Rutgers and Seeda 50, and bolo maka, while causing intermediate symptoms on tomato cv. Insaf. CLVd LP1-15c4 was also found in tomato plants in the northern region of Thailand (personal communication, Dr. Kanungnit Reanwarakorn, Kasetsart University), whereas Solanum-1 was found in bolo maka in central Thailand [5,15,19]. In addition, several Asian CLVd isolates have been reported to cause serious yield losses in tomato crops [5,13]. This is the first report about the correlation between CLVd genome sequences and symptom severities, and three levels of virulence for CLVd were determined.

Undoubtedly, tomato cv. Rutgers was the most susceptible host for all selected CLVd isolates, showing typical strong pospiviroid symptoms. Tomato cv. Seeda 50 also proved to be susceptible to CLVd, and showed strong typical symptoms. Notably, the specific symptom expression on tomato cv. Seeda 50 was slightly different from that observed on Rutgers; Seeda 50 showed stronger leaf yellowing and necrotic lesions but less leaf epinasty and rugosity. Tomato cv. Insaf was the least susceptible tomato host showing mild to moderate symptoms for all four selected CLVd isolates. Interestingly, bolo maka was susceptible to all Thai CLVd isolates, with strong symptoms that varied according to the isolate and which were very similar to the typical symptoms on tomatoes, such as strong stunting, yellowing, epinasty, rugosity and necrosis (Figure 6). In contrast, Thai round eggplant was a less susceptible host for CLVd, showing mild symptoms for all selected CLVd isolates, such as mild leaf distortion and little stunting. Conversely, chili pepper and bell pepper remained symptomless for all selected CLVd isolates, yet all isolates were able to infect and replicate strongly in pepper over the entire lifespan of the plants. This is similar to most natural ornamental hosts—*Columnea erythrophae*, *Nematanthus wettsteinii*, *Brunfelsia undulata*, *Gloxinia gymnostoma*, *G. nematanthodes*, *G. purpurascens*—which showed no symptoms either [3,8,9,10,12]. Interestingly, CLVd Solanum-1 was the only isolate that succeeded in infecting cucumber, however, at a 50% infection rate with no symptoms. The literature about CLVd infectivity in cucumber is somewhat contradictory (Table A2) [6,7,12,13,14,34]. In hindsight, cucumber is probably not a main natural host of CLVd.

Both the multiple-sequence similarity matrix and phylogenetic analysis indicated that CLVd genome sequences cluster into five groups; a major tomato Asia group (I), an ornamental European group (II), a minor tomato Asia group (III), a major tomato European group (IV) and finally a minor tomato European group (V), which are all lineages with a low percentage sequence similarity, even less than 90% for several isolates. This observation corresponds to the results of the symptom severity assessment, in which CLVd 14A, a European isolate, showed different levels of symptom virulence when compared to the three Asia isolates (Chaipayon-1, Solanum-1 and LP1-15c4—group I) on several plant species such as tomatoes, bolo maka and Thai round eggplant. Based on the current ICTV taxonomic classification criteria, these groups would not belong to the same species [16]. In addition, to date, the taxonomic relationship between the ornamental isolates and the Asian and European tomato isolates are still uncertain [21].

Analysis of the conserved motifs on the V domain showed that most of the distinct CLVd lineages (groups) are characterized by different conserved sequences in the motifs (except the two minor groups, CLVd groups III and V) (Figure 9). This relates to recent findings in a CLVd quasi-species population study across several Solanaceae hosts, which demonstrated a high number of SNPs on P and TR domains among most CLVd progeny variants (mutant populations). In contrast, TL, CCR and V domains showed a limited number of mutations, confirming those regions to be very conserved for this viroid [24]. Therefore, it is almost impossible to create a new variant that surpasses its own lineage, due to the relatively wide genomic distance between the CLVd main lineages. This indicates that the three main CLVd groups observed, the main Asian, ornamental and major tomato European lineages (CLVd groups I, II and IV, respectively), possibly evolved from a very early evolutionary split. The two minor tomato Asian and European groups (CLVd groups III and V) possibly evolved from the same evolutionary route, since these two groups shared several similar features, such as genome sequence similarity and the conserved motif on the V domain. In hindsight, the CLVd group III (minor Asia lineage) might possibly be a bridge (or intermediate) group between group V and the main three CLVd groups. In addition, the main Asia lineage (group I) meets the two distinct mandatory criteria for taxonomic reclassification—low sequence similarity and especially different biological properties (symptom expression, disease virulence and yield loss).

For these reasons, we propose that CLVd should be reclassified into at least three main taxonomic lineages—a “CLVd-tomato Asian lineage” (I), a “CLVd-tomato European lineage” (IV) and a “CLVd-ornamental European lineage” (II), plus two minor lineages (III and V). This is similar to several viroid species that have been assigned into several strains by ICTV, such as PLMVd, CEVd, PSTVd, HpSVd, CCCVd, ASSVd and CDVd [16]. In addition, one could argue that these groups could possibly be designated as three different viroid species within the *Pospiviroid* genus. This proposed CLVd taxonomic reclassification fits with the two mandatory criteria required by ICTV; (1) an arbitrary level of less than 90% sequence similarity and (2) distinct biological properties, particularly symptom virulence and severity.

## 5. Conclusions

In this work, we propose the reclassification of the species *Columnea latent viroid* into at least three major lineages, namely, the “CLVd-tomato Asian” (severe isolates originally found in tomato crops in Thailand), “CLVd-ornamental European” (mild isolates originally found in ornamentals) and “CLVd-tomato European” (mild isolates originally found in tomato crops in European countries) lineages. In addition, we identified two minor lineages (corresponding to groups III and V in the phylogenetic study; Figure 7 and Figure 8) containing mild isolates from Thailand and comprising mild isolates from European countries, respectively, of which the latter has been identified as the most distinct lineage, even meriting reclassification into a new viroid species, according to the ICTV criteria.

## Figures and Tables

**Table 1:** CLVd isolates used in this work.

Isolates	Origin	Type of Inocula	Accession No.
14A (93007481)	Tomato—The Netherlands	*S. stramonifolium* (petiole)	AY372392
Chaipayon-1	Tomato—Thailand	*S. stramonifolium* (petiole)	KM214216
LP1-15c4	Tomato—Thailand	*S. stramonifolium* (petiole)	JF446920
Solanum-1	Bolo maka—Thailand	*S. stramonifolium* (petiole)	JF742632

**Table 2:** Symptom rating scale matrix for tomatoes (cvs. Rutgers, Seeda 50 and Insaf) across four CLVd isolates.

Symptoms	Scale						Weight of Score	Score	Score × Weight
	0	1	2	3	4	5			
**Yellowing of Leaves (chlorosis)**	no symptoms	yellowing of leaves (chlorosis): mild	yellowing of leaves (chlorosis): moderate	yellowing of leaves (chlorosis): severe	bronzing and/or purpling: mild	bronzing and/or purpling: severe	10		
**Necrotic Lesions**	no symptoms	mild (midrib or vein)	mild (midrib, vein, petiole and stem)	moderate necrotic lesions	severe necrotic lesions	dieback	40		
**Brittle and Distorted Leaves**	no symptoms	leaves are smaller, bunched together	down-curled (mild: only top of the plant)	down-curled (severe: whole plant)	brittle and distorted (mild)	brittle and distorted (severe)	10		
**Stunted Shoots**	no symptoms	Leaves are smaller	bunched together	Internodes are shortened (mild)	Internodes are shortened (severe)	shoots appear stunted overall	20		
**Stunted Stem**	no symptoms	less than 10% reduction	10%–20% reduction	20%–30% reduction	30%–40% reduction	more than 40% reduction	20		
**Total Score**									

**Table 3:** Symptom rating scale matrix for bolo maka, Thai round eggplant, chili pepper and bell pepper across four CLVd isolates.

Symptoms	Scale						Weight of Score	Score	Score × Weight
	0	1	2	3	4	5			
**Distorted Leaves**	no symptoms	leaves are smaller	rugosity on leaf margin	mild leaf rugosity and epinasty	severe leaf rugosity and epinasty (top part of the plant)	severe leaf rugosity and epinasty (whole plant)	30		
**Necrotic Lesions**	no symptoms	mild (midrib or vein)	moderate (midrib or vein)	severe (midrib or vein)	necrotic lesions at petiole, stem and other parts (mild)	necrotic lesions at petiole, stem and other parts (severe)	40		
**Stunted Stem**	no symptoms	10%–20% reduction	20%–30% reduction	30%–40% reduction	40%–50% reduction	more than 50% reduction	30		
**Total Score**									

**Table 4:** Pairwise identity matrix (%) of four CLVd isolates created by Clustal2.1 (https://www.ebi.ac.uk/Tools/msa/clustalo/, accesed on 30 January 2020).

		14A (93007481)	Chaipayon-1	LP1-15c4	Solanum-1
CLVd isolates	Acc No.	AY372392	KM214216	JF446920	JF742632
**14A (93007481)**	AY372392	100.00	92.82	91.18	92.03
**Chaipayon-1**	KM214216	92.82	100.00	98.36	98.64
**LP1-15c4**	JF446920	91.18	98.36	100.00	98.63
**Solanum-1**	JF742632	92.03	98.64	98.63	100.00

**Table 5:** Total symptom rating scores of four selected CLVd isolates in tomato cultivars Rutgers, Seeda 50 and Insaf, and on bolo maka and Thai round eggplant. (See Table 2 and Table 3 for details about the symptom rating scale used. For more detailed information on symptoms, disease severity and symptom rating scales for each host plant, please see Table A3, Table A4, Table A5, Table A6 and Table A7).

	Symptom Rating Scales				
CLVd Isolates	Tomato			Bolo Maka	Thai Round Eggplant
	Rutgers	Seeda 50	Insaf		
**14A (93007481)**	3.2	2.6	1.1	0.9	0.3
**Chaipayon-1**	4.8	3.8	2.8	3.4	0.6
**LP1-15c4**	5.0	4.5	2.8	4.6	1.3
**Solanum-1**	5.0	4.5	2.8	4.6	1.3

**Table 6:** Overall symptom expression of four selected CLVd isolates in tomato cultivars Rutgers, Seeda 50 and Insaf, and on bolo maka, Thai round eggplant, chili pepper, bell pepper and cucumber.

	Main Symptoms						
CLVd Isolates	Tomatoes			Bolo Maka	Thai Round Eggplant	Chili Pepper and Bell Pepper	Cucumber
	Rutgers	Seeda 50	Insaf				
**14A (93007481)**	- Moderate necrosis - Mild leaf distortion - Severe stunting	- Severe necrosis (black stripe) - Very mild leaf distortion - Moderate stunting	- Very mild necrosis - Very mild leaf distortion - Very mild stunting	- Very mild leaf distortion - Mild stunting	- Very mild leaf distortion	No symptoms	No infectivity
**Chaipayon-1**	- Severe necrosis - Severe leaf distortion - Very severe stunting	- Very severe necrosis (black stripe) - Mild leaf distortion - Moderate stunting	- Severe necrosis - Moderate leaf distortion - Very mild stunting	- Severe necrosis - Moderate leaf distortion - Moderate stunting	- Very mild leaf distortion - Very mild stunting	No symptoms	No infectivity
**LP1-15c4**	- Severe necrosis - Severe leaf distortion - Very severe stunting	- Very severe necrosis (black stripe) - Mild leaf distortion - Severe stunting	- Moderate necrosis - Severe leaf distortion - Very mild stunting	- Severe necrosis - Very severe leaf distortion - Very severe stunting	- Very mild necrosis - Very mild leaf distortion - Very mild stunting	No symptoms	No infectivity
**Solanum-1**	- Severe necrosis - Severe leaf distortion - Very severe stunting	- Very severe necrosis (black stripe) - Mild leaf distortion - Severe stunting	- Moderate necrosis - Severe leaf distortion - Very mild stunting	- Severe necrosis - Very severe leaf distortion - Very severe stunting	- Very mild necrosis - Very mild leaf distortion - Very mild stunting	No symptoms	No symptoms with 50% infectivity

## Appendix A

**Table A1:** 83 Full genome sequences of *Columnea* latent viroid (CLVd) isolates from the NCBI GenBank database (https://www.ncbi.nlm.nih.gov/, accessed on 30 January 2020).

Isolate Names	Accession No.	Original Hosts	Original Countries	Group No.
Chaipayon-1 *	KM214216	*S. lycopersicum*	Thailand	Group I
Chaipayon-16	KM214217	*S. lycopersicum*	Thailand	Group I
Chaipayon-19	KM214218	*S. lycopersicum*	Thailand	Group I
Chaipayon-20	KM214219	*S. lycopersicum*	Thailand	Group I
CR11-15c1	JF446931	*S. lycopersicum*	Thailand	Group I
CR14-9c1	JF446932	*S. lycopersicum*	Thailand	Group I
Duenpen 8	JF742641	*S. lycopersicum*	Thailand	Group I
GC9	AM698093	*S. lycopersicum*	Thailand	Group I
LP1-12c3	JF446917	*S. lycopersicum*	Thailand	Group I
LP1-13c1	JF446918	*S. lycopersicum*	Thailand	Group I
LP1-14c1	JF446919	*S. lycopersicum*	Thailand	Group I
LP1-15c4 *	JF446920	*S. lycopersicum*	Thailand	Group I
LP1-3c1	JF446915	*S. lycopersicum*	Thailand	Group I
LP1-6c5	JF446916	*S. lycopersicum*	Thailand	Group I
LP2-1c1	JF446921	*S. lycopersicum*	Thailand	Group I
LP2-5c1	JF446922	*S. lycopersicum*	Thailand	Group I
LP2-6c1	JF446923	*S. lycopersicum*	Thailand	Group I
LP2-7c1	JF446924	*S. lycopersicum*	Thailand	Group I
LP2-8c1	JF446925	*S. lycopersicum*	Thailand	Group I
LP3-3c2	JF446926	*S. lycopersicum*	Thailand	Group I
LP4-7c1	JF446927	*S. lycopersicum*	Thailand	Group I
LP4-7c4	JF446928	*S. lycopersicum*	Thailand	Group I
LPng16-6c1	JF446933	*S. lycopersicum*	Thailand	Group I
LPng19-4c1	JF446934	*S. lycopersicum*	Thailand	Group I
MC-M-11	AM698095	*S. lycopersicum*	Thailand	Group I
Niruj-14	KM214221	*S. lycopersicum*	Thailand	Group I
Niruj-18	KM214222	*S. lycopersicum*	Thailand	Group I
Niruj-9	KM214220	*S. lycopersicum*	Thailand	Group I
NK10-10c1	JF446935	*S. lycopersicum*	Thailand	Group I
NK10-10c3	JF446936	*S. lycopersicum*	Thailand	Group I
NK11-14c3	JF446938	*S. lycopersicum*	Thailand	Group I
NK11-14c5	JF446939	*S. lycopersicum*	Thailand	Group I
NK11-8c4	JF446937	*S. lycopersicum*	Thailand	Group I
NK-KUKPS1	KY235369	*S. lycopersicum*	Thailand	Group I
PQ-430	DQ923059	*S. lycopersicum*	Thailand	Group I
PQ-49	DQ923058	*S. lycopersicum*	Thailand	Group I
PQ-720	DQ923060	*S. lycopersicum*	Thailand	Group I
PQ-728	DQ923061	*S. lycopersicum*	Thailand	Group I
Prayong-13	KC143293	*S. lycopersicum*	Thailand	Group I
Prayong-16	KC143294	*S. lycopersicum*	Thailand	Group I
Prayong-17	KC143295	*S. lycopersicum*	Thailand	Group I
Rutgers 3	JF742635	*S. lycopersicum*	Thailand	Group I
Rutgers 9	JF742636	*S. lycopersicum*	Thailand	Group I
Sathap-1	KC143289	*S. lycopersicum*	Thailand	Group I
Sathap-2	KC143290	*S. lycopersicum*	Thailand	Group I
Sathap-21	KC143292	*S. lycopersicum*	Thailand	Group I
Sathap-3	KC143291	*S. lycopersicum*	Thailand	Group I
Solanum 1 *	JF742632	*S. stramoniifolium*	Thailand	Group I
Solanum 16	JF742634	*S. stramoniifolium*	Thailand	Group I
Solanum 4	JF742633	*S. stramoniifolium*	Thailand	Group I
CM10-6c1	JF446929	*S. lycopersicum*	Thailand	Group III
CM10-6c2	JF446930	*S. lycopersicum*	Thailand	Group III
NC17	AM698094	*S. lycopersicum*	Thailand	Group III
PC-2-Pa2	DQ022677	*S. lycopersicum*	Thailand	Group III
PC-2-Pa29	DQ061193	*S. lycopersicum*	Thailand	Group III
PC-2-Pa54	DQ061192	*S. lycopersicum*	Thailand	Group III
clone 1-6	JQ362419	*S. lycopersicum*	Mali	Group IV
93007908	AY373446	*S. lycopersicum*	The Netherlands	Group IV
96009271	AY365230	*S. lycopersicum*	The Netherlands	Group IV
93007481 (14A) *	AY372392	*S. lycopersicum*	The Netherlands	Group IV
95001530	AY372395	*S. lycopersicum*	The Netherlands	Group IV
95006685	AY372396	*S. lycopersicum*	The Netherlands	Group IV
FERA_160205	KY810771	*S. lycopersicum*	United Kingdom	Group IV
07060402	FM995506	*S. lycopersicum*	France	Group V
07084401	FM995507	*S. lycopersicum*	France	Group V
89001013	AY367350	*S. lycopersicum*	The Netherlands	Group V
CLVd-IT	HG916813	*S. lycopersicum*	Italy	Group V
EF015581	EF015581	*S. lycopersicum*	Portugal	Group V
12 clone 1	HM043810	*G. purpurascens*	Denmark	Group II
12 clone 2	HM043811	*G. purpurascens*	Denmark	Group II
12 clone 3	HM043812	*G. purpurascens*	Denmark	Group II
12 clone 4	HM043813	*G. purpurascens*	Denmark	Group II
13 clone 1	HM043814	*G. gymnostoma*	Denmark	Group II
13 clone 3	HM043815	*G. gymnostoma*	Denmark	Group II
13 clone 4	HM043816	*G. gymnostoma*	Denmark	Group II
14 clone 1	HM043817	*G. nematanthodes*	Denmark	Group II
14 clone 2	HM043818	*G. nematanthodes*	Denmark	Group II
14 clone 3	HM043819	*G. nematanthodes*	Denmark	Group II
14 clone 4	HM043820	*G. nematanthodes*	Denmark	Group II
CLVd RNA	X15663	*C. erythrophae*	USA	Group II
CLVd(Wild-Type)	AY222072	*C. erythropae*	USA	Group II
complete genome	NC_003538	*C. erythrophae*	USA	Group II
mRNA	M93686	*N. wettsteinii*	Canada	Group II

* = CLVd isolates were used in this work.

**Table A2:** CLVd infectivity on cucumber reported in the literature.

CLVd Isolates	Accession No.	Infectivity on Cucumber	Group No.	References
CLVd NC_003538	NC_003538	Symptomatic (undescribed)	Group II	[12]
CLVd RNA	X15663	Symptomatic (undescribed)	Group II	[12]
CLVd (Wild-Type)	AY222072	well replicated	Group II	[34]
Chaipayon-1 *	KM214216	no infectivity	Group I	This thesis
Solanum 1 *	JF742632	50% infectivity but no symptoms	Group I	This thesis
LP1-15c4 *	JF446920	no infectivity	Group I	This thesis
93007481 (14A) *	AY372392	no infectivity	Group IV	This thesis
PC-2-Pa2	DQ022677	no infectivity	Group III	[14]
PC-2-Pa29	DQ061193	no infectivity	Group III	[14]
PC-2-Pa54	DQ061192	no infectivity	Group III	[14]
MC-M-11	AM698095	replicated but no symptoms	Group I	[13]
NC17	AM698094	replicated but no symptoms	Group III	[13]
GC9	AM698093	replicated but no symptoms	Group I	[13]
93007908	AY373446	No results or details given	Group IV	[7]
95006685	AY372396	No results or details given	Group IV	[7]
95001530	AY372395	No results or details given	Group IV	[7]
89001013	AY367350	No results or details given	Group V	[7]
96009271	AY365230	No results or details given	Group IV	[7]
NK-KUKPS	KY235369	No results or details given	Group I	[6]

* = CLVd isolates used in this work.

**Table A3:** Symptoms, severity and symptom rating scales of four selected CLVd isolates in tomatoes cv. Rutgers.

Symptoms	Symptom Rating Scales			
	14A	Chaipayon-1	Solanum-1	LP1-15c4
Yellowing of leaves (chlorosis)	20	30	50	50
Necrotic lesions	120	200	200	200
Brittle and distorted leaves	20	50	50	50
Stunted shoots	80	100	100	100
Stunted stem (height)	80	100	100	100
**Total Score**	3.2	4.8	5.0	5.0

**Table A4:** Symptoms, severity and symptom rating scales of four selected CLVd isolates in tomatoes cv. Seeda 50.

Symptoms	Symptom Rating Scales			
	14A	Chaipayon-1	Solanum-1	LP1-15c4
Yellowing of leaves (chlorosis)	10	40	30	30
Necrotic lesions	160	200	200	200
Brittle and distorted leaves	10	20	20	20
Stunted shoots	20	60	100	100
Stunted stem (height)	60	60	100	100
**Total Score**	2.6	3.8	4.5	4.5

**Table A5:** Symptoms, severity and symptom rating scales of four selected CLVd isolates in tomatoes cv. Insaf.

Symptoms	Symptom Rating Scales			
	14A	Chaipayon-1	Solanum-1	LP1-15c4
Yellowing of leaves (chlorosis)	10	30	40	40
Necrotic lesions	40	160	120	120
Brittle and distorted leaves	20	30	40	40
Stunted shoots	20	40	60	60
Stunted stem (height)	20	20	20	20
**Total Score**	1.1	2.8	2.8	2.8

**Table A6:** Symptoms, severity and symptom rating scales of four selected CLVd isolates in bolo maka.

Symptoms	Symptom Rating Scales			
	14A	Chaipayon-1	Solanum-1	LP1-15c4
Distorted leaves	30	90	150	150
Necrotic lesions	0	160	160	160
Stunted stem (height)	60	90	150	150
**Total Score**	0.9	3.4	4.6	4.6

**Table A7:** Symptoms, severity and symptom rating scales of four selected CLVd isolates in Thai round eggplant.

Symptoms	Symptom Rating Scales			
	14A	Chaipayon-1	Solanum-1	LP1-15c4
Distorted leaves	30	30	60	60
Necrotic lesions	0	0	40	40
Stunted stem (height)	0	30	30	30
**Total Score**	0.3	0.6	1.3	1.3
